# Dose-Levels and First Signs of Efficacy in Contemporary Oncology Phase 1 Clinical Trials

**DOI:** 10.1371/journal.pone.0016633

**Published:** 2011-03-02

**Authors:** Charles Ferte, Jean-Charles Soria, Nicolas Penel

**Affiliations:** 1 Département de Cancérologie Générale, Centre Oscar Lambret, Lille, France; 2 SITEP (Phase 1 unit), Département de Médecine, Institut Gustave Roussy, Villejuif, France; 3 Equipe d’Accueil 2694: Santé Publique, Epidémiologie et modélisation des maladies chroniques, Université Lille II, Lille, France; Institute for Medical Biomathematics, Israel

## Abstract

**Purpose:**

Phase 1 trials play a crucial role in oncology by translating laboratory science into efficient therapies. Molecular targeted agents (MTA) differ from traditional cytotoxics in terms of both efficacy and toxicity profiles. Recent reports suggest that higher doses are not essential to produce the optimal anti-tumor effect. This study aimed to assess if MTA could achieve clinical benefit at much lower dose than traditional cytotoxics in dose seeking phase 1 trials.

**Patients and Methods:**

We reviewed 317 recent phase 1 oncology trials reported in the literature between January 1997 and January 2009. First sign of efficacy, maximum tolerated dose (MTD) and their associated dose level were recorded in each trial.

**Results:**

Trials investigating conventional cytotoxics alone, MTA alone and combination of both represented respectively 63.0% (201/317), 23.3% (74/317) and 13.7% (42/317) of all trials. The MTD was reached in 65.9% (209/317) of all trials and was mostly observed at the fifth dose level. First sign of efficacy was less frequently observed at the first three dose-levels for MTA as compared to conventional cytotoxics or combinations regimens (48.3% versus 63.2% and 61.3%). Sign of efficacy was observed in the same proportion whatever the treatment type (73–82%). MTD was less frequently established in trials investigating MTA alone (51.3%) or combinations (42.8%) as compared to conventional cytotoxic agents (75.6%).

**Conclusion:**

First sign of efficacy was less frequently reported at the early dose-levels and MTD was less frequently reached in trials investigating molecular targeted therapy alone. Similar proportion of trials reported clinical benefit.

## Introduction

Phase 1 oncology trials play a crucial role in translating laboratory science into effective therapies. These trials enroll patients with advanced cancer that have mostly exhausted all available standard care, in order to evaluate the safety profile and the pharmacokinetic properties of new therapeutic regimens. The primary aim of such studies remains to establish the optimal recommended dose for further trials. Traditional phase 1 oncology trials consist in the administration of increasing doses of the experimental compound to successive cohorts of patients until the maximal tolerated dose is reached. This design was developed in the context of the conventional cytotoxic agents, which exhibit dose-efficacy and dose-toxicity relationships. The highest dose patients could tolerate was thought to produce the greatest benefit [1]–[2]. Therefore, the conventional metric and endpoint to determine the optimal phase 2 recommended dose is the experienced toxicity [3].

The recent increase in the knowledge of molecular mechanisms implicated in cancer growth led to the emergence of a vast number of novel therapeutic agents (i.e.: monoclonal antibodies, tyrosine kinase inhibitors, proteasome inhibitors, demethylating agents, pro-apoptotic agents…). This new class of agents demonstrated very different toxicity and efficacy profiles compared to traditional cytotoxics. It has been advocated that MTD would not be the best approach to generate the maximum anti-tumor effect [4]–[5]. Molecular targeted agents might then achieve clinical benefit at lower dose levels than those required with conventional cytotoxic agents. To further gain insight on this topic, we reviewed 317 recent phase 1 oncology trials, in order to evaluate if the clinical benefit is observed at lower doses with molecular targeted therapies compared to conventional cytotoxic agents.

## Materials and Methods

### Selection of publications

The reports considered for the present study were found by searches of Medline using the terms: “clinical trial, phase I”, “solid tumors”. References were selected from the following oncology journals that usually deal with phase I trials: *Annals of Oncology*, *European Journal of Cancer*, *Journal of Clinical Oncology, Clinical Cancer Research and Investigational New Drugs*. Only articles published in English between 1997, January and 2009, January were included. The most frequently tested agents were identified and assessed for relevance by use of the following parameters: Only phase 1 studies investigating systemic treatments with dose seeking were selected, whereas phase 1–2 trials, radiation therapy trials, intra-tumoral or loco-regional treatments (e.g. intra-peritoneal injections) trials and organ impairment studies were excluded. A total of three hundred and five publications reporting 317 dose-seeking phase-I-trials were finally herein analyzed.

### Data extraction and definitions

The following pieces of information were extracted: date of publication, type of treatments (cytotoxic agents, molecular-targeted agent and combination of both), number of explored dose-levels, dose-level associated with maximal tolerated dose and dose level associated with first signs of efficacy. The maximal tolerated dose (MTD) was defined as the dose where a pre-specified number of patients (usually 2 of 3 or 2 of 6) exhibits dose-limiting toxicity. The first evaluation was mostly scheduled after two cycles of treatment. Sign of efficacy was defined as complete response (CR) or partial response (PR) as well as stable disease (SD) (according to validated criteria such as RECIST or WHO) or long-lasting stable disease (defined as either CR or PR or SD > 3 months [6]).

### Statistical analysis

The description of the results was based on classical statistical methods: median and range, mean and standard derivation, percentage and 95%-confidence intervals (95%-CI). Comparisons used Mann-Whitney test for continuous variables and Fischer exact test for categorical variables. P-values were done when the test had shown a significant difference (p<0.05). The collected data were analyzed using SPSS version 13.0 statistical software.

## Results

### General

**Figure 1:**
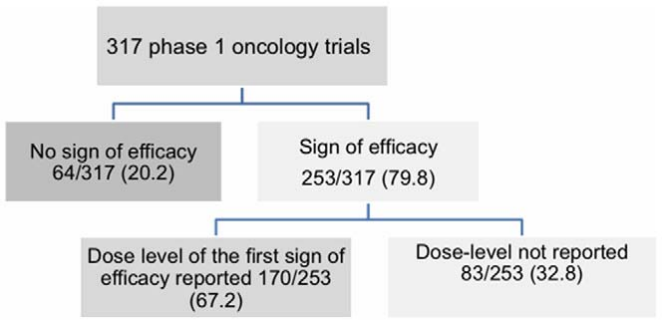
Sign of efficacy and dose levels in phase 1 oncology trials.

The sample included the results of 317 phase 1 oncology trials published between January 1997 and January 2009 (Figure 1). Trials of conventional cytotoxic agent(s) alone accounted for 63.0% (201/317) of all trials. Trials investigating molecular targeted therapies alone represented 23.3% (74/317) of all trials. Trials investigating a combination of conventional cytotoxic agents and molecular targeted therapies accounted for 13.7% (42/317) of all trials. The MTD was established in 65.9% (209/317) of all trials. The formal MTD was not established at the end of the trial in 25.2% of all studies (80/317). No clear MTD data was reported in 28 reports (8.9%). The median number of tested dose-levels was five in all treatment categories (Table 1). In most cases, MTD was reached at the fifth dose level. A total of 253/317 trials (79.8%) exhibited signs of drug activity. The dose level associated with first sign of efficacy was reported in 170/253 trials (67.2%): median 1, range 1–9.

**Table 1:** Sign of efficacy and MTD in 317 phase 1 oncology trials.

	Trials investigating Conventional cytotoxic agents	Trials investigating molecular targeted agents	Trials investigating combination of both
MTD established			
no. (%)	152/201 (75.6) *	38/74 (51.3)	18/42 (42.8)
[95%-CI]	[69.7–81.5]	[39.9–62.7]	[27.8–57.8]
Dose-level associated with DMT			
Median	5	5	4
Range	1–13	1–16	1–12
Sign of efficacy			
*no*. (%)	165/201 (82.0)	58/74 (78.3)	31/42 (73.8)
[95%-CI]	[76.7–87.4]	[69.8–87.7]	[60.5–87.7]
Dose-level associated with first sign of efficacy			
Median	1	1	1
Range	1–7	1–9	1–3
First sign of efficacy seen at the first dose-level			
*no*. (%)	57/109 (52.2)	15/60 (25.0) *	16/31 (51.6)
[95%-CI]	[42.9–61.6]	[14.0–35.9]	[34.0–69.2]
First sign of efficacy seen at the first 3 dose-levels			
*no*. (%)	81/109 (74.3)	29/60 (48.3) *	19/31 (61.3)
[95%-CI]	[66.1–82.5]	[35.6–60.9]	[44.1–78.4]
First sign of efficacy seen before MTD			
*no*. (%)	91/144 (63.2)	27/44 (61.3)	13/25 (52.0)
[95%-CI]	[55.3–71.0]	[46.9–75.7]	[32.4–71.6]
Sign of efficacy without MTD reached			
*no*. (%)	26/193 (13.4)	10/66 (15.0)	21/32 (65.6) *
[95%-CI]	[8.6–18.3]	[6.5–23.8]	[49.1–82.0]

MTD: dose maximal tolerated dose;

*: p<0.05.

The total of trials within the same treatment categories could differ in the different lines because no data concerning MTD was available in 28 trials and because the first sign of efficacy was not linked to a dose-level in 64 trials.

### Comparison according to treatment categories

MTD was less frequently reached in trials investigating targeted therapies alone (51.3%) or the combinations (42.8%) compared to conventional cytotoxic agents (75.6%) (p<0.0001, Fischer exact test). Sign of efficacy was observed at the same proportion in the 3 treatment categories: 82.0% for conventional cytotoxic agent, 78.3% for molecular targeted therapies and 73.8% for their combinations. First sign of efficacy was observed in most cases at the first or second dose-level. First sign of efficacy was less frequently observed at the first dose-level for molecular targeted therapies (25.0%) compared to conventional cytotoxic agents (52.2%) or their combinations (51.6%) (p<0.0001, Fischer exact test). It was also less frequently observed at the three first dose-levels of trials testing molecular targeted therapies alone (48.3% versus 63.2% and 61.3%) (p<0.0001, Fischer exact test). The first sign of efficacy was observed before the MTD with the same proportion (52%–63.2%) whatever the treatment categories. But, the trials investigating combinations reached sign of efficacy without establishing MTD in the largest proportion as compared to those investigating conventional cytotoxics or molecular targeted agents alone (65% versus 13.4% and 15.0%) (p<0.05).

## Discussion

We observed that first signs of efficacy were less frequently reported at early dose levels in MTA phase 1 trials than in conventional cytotoxics ones. These findings are in apparent contradiction with Postel-Vinay et al. and Jain et al., which described similar rates of clinical benefit whatever the tested dose of the MTA [6]–[7]. Both authors used empiric cut-offs based on the percentage of MTD to partition the patients. Such methodology may be sub-optimal since the escalating dose design is generally not linear (e.g.: Fibonacci escalation). This reason led us to propose a more precise analysis based on the dose level rather than on the percentage of MTD. We hypothesize that the choice of very conservative starting dose and the use of unsuitable dose-escalation rules mainly explain why first signs of efficacy were less frequently observed at early dose-levels in MTA trials. Regarding the starting dose, the translation rules from animal to man are rough (the usual starting dose is calculated based on the tenth of the animal LD10) and no recent studies yet addressed this question regarding the MTA setting [8]–[11]. The Health Authorities also favor very conservative approaches, with very low starting doses. Moreover, the classical dose escalation rules (i.e. Fibonacci escalation, 3+3 design, dose-effect and dose-toxicity relationships) may not be suitable for MTA phase 1 trials. Most of dose-seeking phase 1 trials investigating MTA are designed with the same underlying assumptions as those made for traditional cytotoxics phase 1 trials: (i) a higher dose is preferred, if tolerable, since it would produce more tumor shrinkage [1]–[2] (ii) the main metric to establish the phase-2-recommended dose is then the experienced toxicity [12]–[13]. However, both the absence of dose-effect relationship [6]–[7] and the trend towards long lasting stable disease rather than tumor shrinkage [4]–[5] were observed in the MTA setting. New designs need to be more largely explored, such as accelerated titration design and continual reassessment method, which could allow more rapid dose-escalation and reduce the number of patients treated with infra-therapeutic dose-levels [14].

Our results also pointed out that MTD was less frequently identified in MTA phase 1 trials. The pertinence of establishing MTD for MTA is debatable. On the first hand, the toxicity profile of MTA must be evaluated likewise every investigated agent. Dramatic examples, reminded clinicians that toxicity remains a major endpoint in MTA phase 1 trials [15]–[18]. On the other hand, some recent drugs have been successfully developed without reaching MTD (e.g. Trastuzumab, Rituximab). The optimal dose also remains questionable at late development stages for other drugs (e.g. Sunitinib [19], Sorafenib [20], Imatinib [21]). Some reasons might limit the assessment of MTD in contemporary trials: (i) MTD might never be reached given that some of the MTA's adverse events do not fulfill the traditional DLT criteria (e.g.: hematotoxicity, nausea…) but rather represent “organ-type” toxicities that are difficult to predict [22] (ii) To push the drug dose up to the MTD might not lead to greater clinical or biological effects since MTA could not exhibit dose dependant toxicity or efficacy profiles [6]–[7]. Several biological concepts may explain this fact. For instance, the binding of monoclonal agents to their specific epitopes is a saturable system [23]–[24]. Increasing their dose would not lead to a gain in efficacy when the plateau is reached [23]. Moreover, to increase the dose of protein kinase inhibitors would decrease their specificity and render hazardous the effect they are supposed to play [25]. Finally, both investigators and industrials might opt to prematurely stop the assay before the MTD level in order to limit substantial time and resource consuming process. New biological endpoints (i.e: effective inhibition of the molecular target) have been proposed to supplant classical toxicity endpoints but remain investigational. To date, no biomarker has yet been validated as a consensual endpoint for MTA phase 1 trials. [5]; [26]–[27].

A similar proportion of trials reporting signs of efficacy was herein observed, whatever the treatment type (conventional cytotoxics vs. MTA). This finding is consistent with the landmark 11,000-patient study reported by Horstmann et al, describing similar non progression rates among patients enrolled in phase 1 trials investigating cytotoxic chemotherapy alone (45,2%) and MTA alone: immunomodulator (46,8%), receptor or signal transduction inhibitor (42,4%), antiangiogenic agent (34,9%) [28]. Such similar high clinical benefit rate could however be secondary to the favored enrollment of patients with “low-progressive” disease. Indeed, the huge number of eligibility criteria implies to select patients with very healthy condition, without any comorbidity and with normal liver, renal or hematological functions. The industrial sponsors pressure under investigators could also favor this trend. Recently, pre-treatment growth dynamics were demonstrated to have major impact on the RECIST tumor evaluation [29]. In this context, contemporary phase 1 trials should include the assessment of tumor growth kinetics before the administration of the experimental regimens.

The fact that combination phase 1 trials achieve sign of efficacy before MTD in the largest proportion is not surprising. By these studies, most investigators assume that the adjunction of a new drug (i.e.: molecular targeted agent) will improve the antitumoral effect of an already efficient drug (i.e.: conventional cytotoxic). Then, some characteristics of the companion cytotoxic (i.e.: the nature of the drug confronted to the patient's tumor types, the starting dose and the incremental scheme adopted) could strongly influence their efficacy profile. Unfortunately, this retrospective view does not allow us to correlate the dose response profile with these probable associated factors.

This study presents nevertheless some limitations due to its retrospective nature and the fact we used published data. Our sample of studies is heterogeneous in term of agents investigated and types of cancer. The lack of uniform reporting standard and publication biases may also contribute to misestimate the real clinical benefit. Moreover, our study was not designed to discriminate different subsets among the trials testing molecular targeted agents. For instance, monoclonal antibodies would certainly differ form other agents (e.g.: tyrosine kinase inhibitors, proteasome inhibitors, vascular disrupting agents) due to their particular pharmacological properties. Of note, such assessment is currently ongoing on prospective individual data. Further, the definition of first sign of efficacy could be discussed: results would certainly be different if only CR and PR were taken in account for its definition. However, to consider SD as a proof of efficacy is relevant since a large body of literature reports that MTA rather induce stable disease than tumor volume shrinkage [4]–[5]. Finally, the fact that more and more patients benefit from tumor molecular profiling has to be noticed. Phase 1 trials are then being enriched with tumors harboring the molecular aberrations that are more susceptible to respond to the tested drug (e.g.: the inclusion of tumors with PTEN deletion or PIK3CA mutation in mTOR inhibitors trials). This phenomenon could have an unexpected impact on the dose finding process. For example, exquisite responses could be observed at a very low dose level.

Although the concept of optimal biological dose (OBD) was recently introduced, it has only rarely been used in practice for dose seeking [22]; [26]. Several facts explain this situation. First, pharmacokinetic and pharmacodynamic data are often limited by technical problems and are mostly addressed with delay (i.e.: usually after the recommended dose is found). Secondly, the small amount of patients per dose level renders difficult to correlate the biological surrogate with the drug concentration. Given the very restricted number of published trials using OBD to determine the recommended phase 2 dose, we could not explore if signs of efficacy were observed earlier in these trials.

Signs of efficacy are described in most phase 1 oncology trials. When investigating molecular targeted agents alone, first signs of efficacy are less frequently observed at the early dose levels, and MTD are less frequently reached. The present report points out the necessity to refine the classical designs in the era of molecular targeted therapies. Binary primary endpoints including both tolerability and efficacy (objective response and long-lasting stable disease) are warranted. The implementation of new approaches for choosing the starting dose and of accelerated titration designs could avoid the treatment of patients at under optimal dose.
